# Sol-Gel Synthesis of New Bioactive Organic-Inorganic Materials for Biomedical Use: SiO_2_/Ferulic Acid/PEG

**DOI:** 10.3390/ijms27062698

**Published:** 2026-03-16

**Authors:** Federico Barrino, Federica Giuliano, Harrison de la Rosa-Ramírez, María Dolores Samper

**Affiliations:** 1 Department of Engineering, University of Palermo, Viale delle Scienze 6, 90128 Palermo, Italy; 2 Institute of Materials Technology (IUTM), Universitat Politècnica de València (UPV), 46022 Alicante, Spain

**Keywords:** sol-gel, ferulic acid, natural drug, PEG

## Abstract

In this study, a series of SiO_2_-based biomaterials synthesized via the sol-gel technique was developed by integrating different weight percentages (10_wt_% and 15_wt_%) of ferulic acid (FA) and varying weight percentages (6_wt_%, 12_wt_%, and 24_wt_%) of polyethylene glycol (PEG). Chemical characterization of the materials was performed by FTIR-ATR spectroscopy to confirm the incorporation of the functional agents and the matrix structure. Biocompatibility was assessed through cell-based assays and gene expression analysis, highlighting a positive effect of the materials on cell proliferation and the regulation of key markers for tissue regeneration. Finally, the ability to induce hydroxyapatite (HA) formation was verified using simulated body fluid (SBF) following the Kokubo test, demonstrating the bioactive potential of the treated surfaces. The obtained results indicate that the combination of SiO_2_, FA, and PEG via sol-gel represents a promising platform for applications in the field of bone regeneration and functional biomaterials.

## 1. Introduction

In recent years, the field of tissue engineering has emerged as a central discipline in regenerative medicine, aiming to develop advanced strategies to repair, replace, or regenerate damaged tissue [1,2]. In this field, the utilization of biomaterials is paramount, as they provide both structural support and biochemical signals capable of stimulating cell proliferation and differentiation [3]. A fundamental objective of contemporary biomedical research is the conception of multifunctional biomaterials that efficaciously integrate biocompatibility, biodegradability, and therapeutic activity [4,5].

Among the most promising materials for regenerative applications are silicon dioxide (SiO_2_)-based biomaterials, which are valued for their extraordinary chemical and physical properties, structural stability, and ability to interact favorably with the biological environment. In particular, materials obtained through the sol-gel process offer the possibility of integrating water-soluble polymers and bioactive molecules within the inorganic silica network, giving rise to highly versatile and customizable organic-inorganic hybrid systems [6]. The sol-gel technique is a method of synthesizing these materials that is performed at low temperatures and under controlled chemical conditions. The process commences with alkyl silane precursors, such as tetraethyl orthosilicate (TEOS), and progresses through successive hydrolysis and condensation reactions. These reactions result in the formation of a three-dimensional silica network [7].

The incorporation of bioactive organic components represents a well-established methodology for enhancing the functionality of sol-gel materials [8]. Among these, FA, a naturally occurring phenolic molecule, is of particular interest for medical applications due to its antioxidant, antibacterial, and anti-inflammatory properties [9,10]. However, for these properties to be maintained over time, it is essential that FA be integrated effectively and stably into the inorganic matrix, preserving its bioactivity [11].

Another organic component of significant relevance is PEG, which is notable for its high biocompatibility and hydrophilicity, as well as its ability to modulate the material’s physical characteristics, such as porosity, flexibility, and controlled release of active substances [12]. The incorporation of PEG into sol-gel materials has been demonstrated to exert a favorable influence on matrix degradation and cellular interaction, thereby fostering the establishment of a microenvironment that is conducive to tissue regeneration. Furthermore, the weight percentage of PEG utilized during synthesis can significantly impact the microstructure of the final material, resulting in variations in cross-link density and the release kinetics of bioactive compounds [13,14].

From the results of the scientific work of Barrino et al. (2025) [11], the best weight percentages of hybrid materials based on silicon and ferulic acid were chosen. The latter are glassy materials that are difficult to use in the biomedical field as coatings for existing prostheses. For this reason, the new study aims to improve the properties of glassy materials by adding a polymer to reduce their fragility.

In view of these findings, the investigation of SiO_2_-based sol-gel biomaterials that have been functionalized with varying weight percentages of PEG (6_wt_%, 12_wt_%, and 24_wt_%) and the best FA (10_wt_% and 15_wt_%) is a promising approach for the development of high-performance systems that can meet the complex requirements of regenerative medicine. A crucial step in the rational design of new biomedical scaffolds or coatings, including those intended for integration with existing prostheses, is a systematic analysis of how these two components influence the chemical, physical, and biological characteristics of the final material.

The objective of this study is therefore to synthesize and characterize organic-inorganic hybrid materials using the sol-gel process, evaluating the impact of varying concentrations of PEG and FA on the structure and functional properties of the resulting materials. The anticipated outcomes could facilitate the creation of novel biocompatible and multifunctional systems, which hold considerable promise for utilization in regenerative medicine, particularly as scaffolds for supporting cell proliferation, inducing gene expression, and the targeted release of bioactive molecules.

## 2. Results

### 2.1. Sol-Gel Biomaterials

The biomaterials were synthesized using the sol-gel technique, employing TEOS as an inorganic precursor to form the silicon dioxide (SiO_2_) matrix. FA and PEG were incorporated into the sol-gel network at varying weight percentages. Six samples were obtained, and the formulations are shown in Table 1. All materials appeared as homogeneous, stable, and glassy xerogels, with no apparent phase separation or precipitation of organic components. The addition of FA and PEG did not interfere with the silane hydrolysis and condensation process, allowing the formation of a continuous three-dimensional silica network. It was observed that as the PEG content increased, the samples became more transparent and less rigid in consistency, suggesting a plasticizing effect of the polymer on the sol-gel structure. Furthermore, formulations with 15_wt_% FA showed a slightly more intense amber color than samples with 10_wt_%, consistent with the increased phenolic loading. The solid-state mass recovery for all samples was high (>90%), with gelation times varying between 24 and 72 h at room temperature, primarily influenced by the PEG concentration. Indeed, as PEG content increased, gelation times tended to become longer, likely due to the steric interaction of the polymer with the silane groups.

### 2.2. FTIR-ATR Analysis

**Figure 1:**
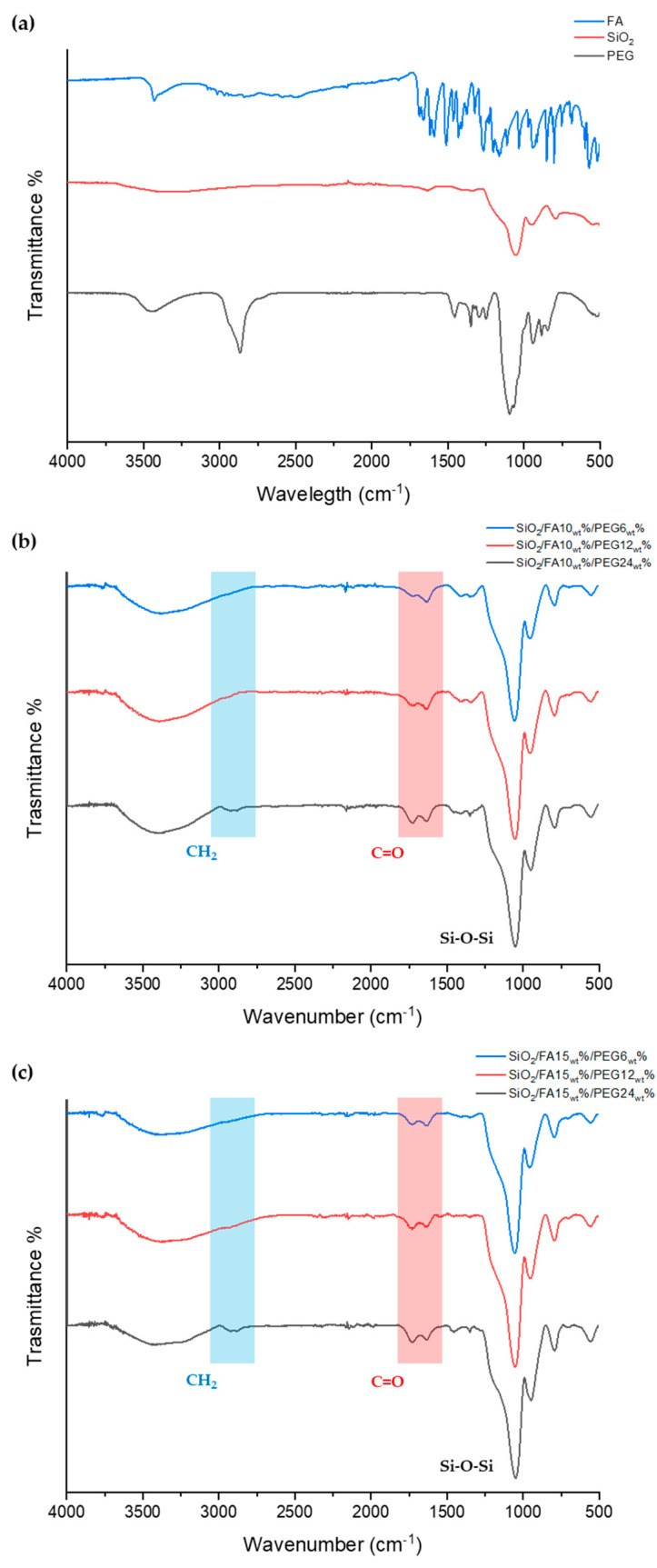
FTIR-ATR spectra of: (**a**) pure natural drug (FA), silica matrix (SiO_2_), and polyethylene glycol (PEG). FTIR-ATR spectra of synthesized hybrid materials: (**b**) SiO_2_/FA10_wt_%/PEG6_wt_%, SiO_2_/FA10_wt_%/PEG12_wt_%, and SiO_2_/FA10_wt_%/PEG24_wt_%, and (**c**) SiO_2_/FA15_wt_%/PEG6_wt_%, SiO_2_/FA15_wt_%/PEG12_wt_%, and SiO_2_/FA15_wt_%/PEG24_wt_%.

To correctly understand and interpret the spectra of the hybrid materials obtained via sol-gel, FTIR-ATR spectroscopic analysis of the individual components was first performed and is reported in Figure 1a.

SiO_2_ exhibits a spectrum typical of amorphous silica-based materials. A very intense, broad band centered around 1080 cm^−1^ is observed, attributable to the asymmetric stretching motion of the Si–O–Si bond, which represents the most representative vibration of the three-dimensional silica network. At lower frequencies, a second band is detected around 800 cm^−1^, corresponding to the symmetric Si–O–Si stretching motion. A signal around 950 cm^−1^ is commonly attributed to the presence of terminal Si–OH groups (silanols), a characteristic of xerogels. Furthermore, in the 3400 cm^−1^ region, a broad band is observed due to the stretching of the OH groups, both from silanols and from adsorbed water [15,16].

PEG, a hydrophilic linear polymer composed of repeating ether units, exhibits a well-defined and easily recognizable spectrum. In the region of 2880–2920 cm^−1^, the stretching band of the C–H bonds of the methylene groups CH_2_ along the polymer chain is observed. The most intense band, however, is located between 1100 and 1145 cm^−1^ and is attributed to the stretching of the C–O–C ether bridge. Other minor but significant signals are found around 1460 cm^−1^ (CH bending) and 1340 cm^−1^ (CH_2_ bending) [17].

The spectrum of FA, a conjugated phenolic compound with antioxidant activity, exhibits a complex set of characteristic bands. In the high region of the spectrum (~3500–3250 cm^−1^), a broad signal is present, attributable to the stretching of the phenolic hydroxyl group (O–H), often partially overlapping with the water band. The carboxylic acid group is well represented by a sharp and intense band between 1690 and 1715 cm^−1^, corresponding to the stretching of the C=O bond. The aromatic system displays several C=C stretching bands in the range between 1600 and 1450 cm^−1^, while the contribution of the C–O stretching of the phenolic group is detected in the region between 1240 and 1260 cm^−1^.

Overall, the FTIR-ATR spectra of the three components show distinctive signals that allow for the precise identification of each constituent and its monitoring within the subsequently obtained hybrid matrices.

FTIR-ATR spectroscopic analysis of the hybrid materials obtained by sol-gel synthesis confirmed the effective incorporation of FA and PEG within the inorganic silicon dioxide matrix (Figure 1b,c), with evidence of chemical interaction between the components. The spectra show characteristics attributable to all three components, with variations depending on their concentration. A broad and intense band centered around 1080 cm^−1^ is observed in all samples, attributable to the asymmetric stretching of the Si–O–Si bond, typical of the three-dimensional amorphous silica network. This band constitutes the main contribution of the inorganic backbone. The peak at approximately 950 cm^−1^, indicative of free silanol groups (Si–OH), is present in all samples, suggesting partial condensation of the sol-gel network, influenced by the presence of organic components.

With the addition of PEG, a progressive increase in band intensity is observed in the 1100–1145 cm^−1^ region, consistent with the contribution of the stretching of the C–O–C ether bridge characteristic of the polymer. This increase is clearly correlated with the weight concentration of PEG. Samples with 12_wt_% and especially 24_wt_% show stronger signals than those with 6_wt_%, confirming the increasing presence of the polymer in the matrix. In parallel, the bands associated with the CH_2_ methylene groups (stretching at ~2880–2920 cm^−1^ and bending at ~1465 cm^−1^) progressively increase with the amount of PEG introduced, visible mainly at 24_wt_%.

The incorporation of FA is confirmed by the presence of the carbonyl band (C=O) of the carboxylic acid group, located around 1710–1690 cm^−1^, which is not present in pure silica. This band becomes more intense in samples containing 15_wt_% FA compared to those with 10% FA, indicating a higher content of the phenolic compound. The signal at approximately 1240–1260 cm^−1^, attributable to C–O stretching of the phenolic group, also appears progressively more intense in FA-enriched samples. This trend is consistent with a homogeneous distribution of the active ingredient in the matrix and confirms its structural integration within the sol-gel network [18,19]. This is further supported by a slight shift in the carbonyl band of FA, indicating a hydrogen-bonding interaction with the silanol groups of the silica matrix.

The FTIR-ATR spectra of the hybrid materials show a well-defined combination of characteristic signals from SiO_2_, PEG, and FA, with a gradual, proportional evolution of the peaks associated with the organic components. The presence and increase in intensity of the PEG and FA bands, as a function of their concentration, represent a further confirmation of the successful incorporation of the functional components into the inorganic matrix.

### 2.3. Biocompatibility

#### 2.3.1. Citocompatibility

**Figure 2:**
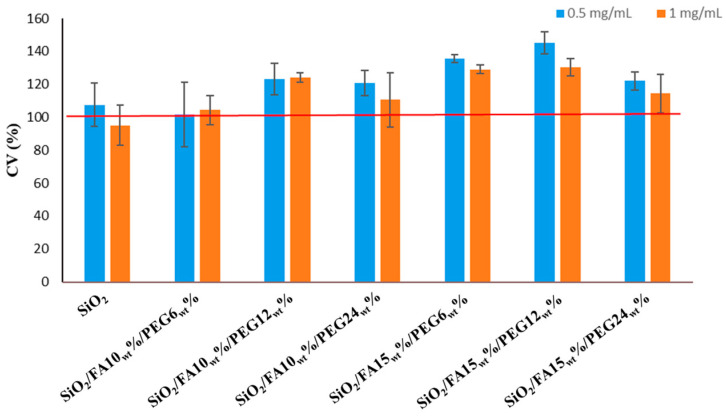
Cytocompatibility of the synthesized materials compared with the CTR (SiO_2_) at two different dosages (0.5 and 1.0 mg/mL).

Figure 2 shows that none of the samples was cytotoxic toward HDF cells; on the contrary, all formulations maintained or improved cell viability. The independent-sample t-test, *p* < 0.05, displayed that at 0.5 mg/mL, a significant increase in cell viability compared to CTR was found for all samples except SiO_2_ and SiO_2_/FA10_wt_%/PEG12_wt_%. At 1 mg/mL, there was a significant increase vs. CTR in HDF viability for SiO_2_/FA10_wt_%/PEG12_wt_%, SiO_2_/FA15_wt_%/PEG6_wt_%, and SiO_2_/FA15_wt_%/PEG12_wt_%. However, when comparing the two dosages, it was found that the lower one showed the most favorable trend in cell viability, with the highest values recorded for SiO_2_/FA15_wt_%/PEG6_wt_% and SiO_2_/FA15_wt_%/PEG12_wt_% (135% and 145% vs. CTR (100%)).

#### 2.3.2. Gene Expression

**Figure 3:**
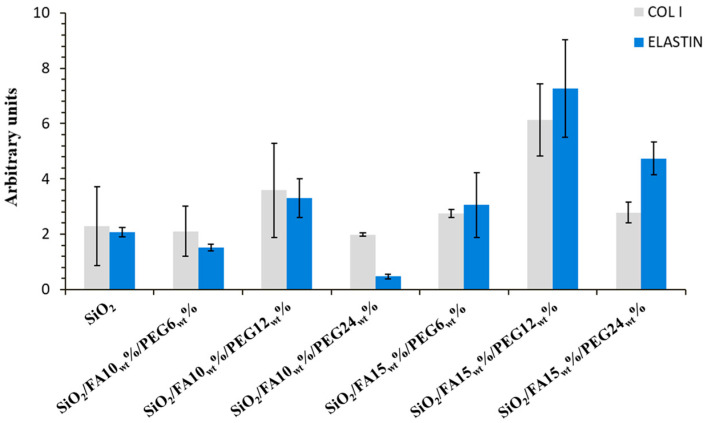
Gene expression of COL I and ELASTIN compared to CTR.

Figure 3 displayed that all the samples increased the gene expression of both COL I and ELS with respect to CTR, except for SiO_2_/FA10_wt_%/PEG6_wt_% and SiO_2_/FA10_wt_%/PEG24_wt_% materials. It is interesting to note that the biomaterial containing 12% of PEG resulted in better performance compared to the others. In detail, SiO_2_/FA10_wt_%/PEG12_wt_% and SiO_2_/FA15_wt_%/PEG12_wt_% up-regulated vs. CTR the gene expression of COL I by about 3.5 and 6.1 fold. Concerning ELS modulation, beyond SiO_2_/FA10_wt_%/PEG12_wt_% and SiO_2_/FA15_wt_%/PEG12_wt_%, SiO_2_/FA15_wt_%/PEG24_wt_% also proved a good efficacy in increasing it. ELS gene expression was up-regulated by about 3.3, 7.6 and 4.7 fold, respectively, in SiO_2_/FA10_wt_%/PEG12_wt_%, SiO_2_/FA15_wt_%/PEG12_wt_% and SiO_2_/FA15_wt_%/PEG24_wt_%.

### 2.4. Bioactivity

#### Apatite-Structure Formation

**Figure 4:**
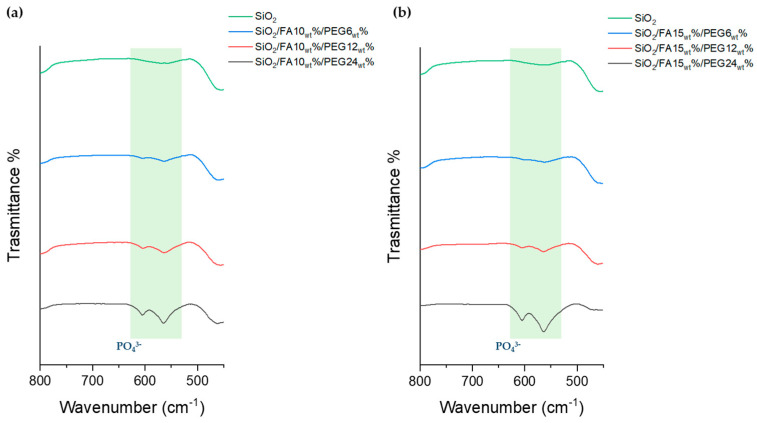
FTIR-ATR spectra of all hybrid materials at FA10_wt_% (**a**) and FA15_wt_% (**b**) compared with SiO_2_ after 21 days of immersion in SBF solution.

The bioactivity of sol-gel-derived hybrid materials was investigated through FTIR-ATR spectroscopy following 21 days of immersion in SBF (Figure 4). This approach enables the evaluation of a material’s ability to form a bone-like apatite layer on its surface [20]. The collected FTIR-ATR spectra revealed a progressive and composition-dependent development of phosphate bands, centered in the 560–600 cm^−1^ region, corresponding to the bending modes of PO_4_^3−^ groups typically found in HA. Notably, these bands were completely absent in the reference SiO_2_ spectrum, confirming that the unmodified silica network lacks sufficient bioactivity under the tested conditions. In contrast, all hybrid samples containing FA and PEG displayed varying degrees of phosphate signal intensity, clearly indicating successful nucleation and growth of a calcium phosphate phase. A clear compositional trend was observed: within each FA series, increasing the PEG content, from 6_wt_% to 24_wt_%, led to more intense and better-defined phosphate bands, while at the same PEG content, the 15_wt_% FA systems generally showed a stronger response than the 10_wt_% FA systems. These findings indicate that both PEG and FA influence the apatite-forming ability of the hybrids. However, the present data do not directly demonstrate the release of PEG or FA during SBF immersion; therefore, their role should be interpreted cautiously. A plausible explanation is that PEG, because of its hydrophilic character, increases water uptake and facilitates the penetration of the SBF solution into the hybrid matrix. This may enhance ion diffusion and promote the exposure of silanol-rich interfacial sites, which are known to favor calcium phosphate nucleation. FA may also contribute by modifying the local chemical environment and the structural organization of the hybrid network through interactions with the silica matrix. In this view, the enhanced apatite formation observed in PEG and FA-containing samples is more reasonably attributed to changes in matrix hydration, permeability, and surface reactivity than to phosphate formation alone. The most bioactive formulations were clearly SiO_2_/FA10_wt_%/PEG24_wt_% and SiO_2_/FA15_wt_%/PEG24_wt_%, which showed the highest intensity of PO_4_^3−^ bands, indicating extensive mineralization.

**Figure 5:**
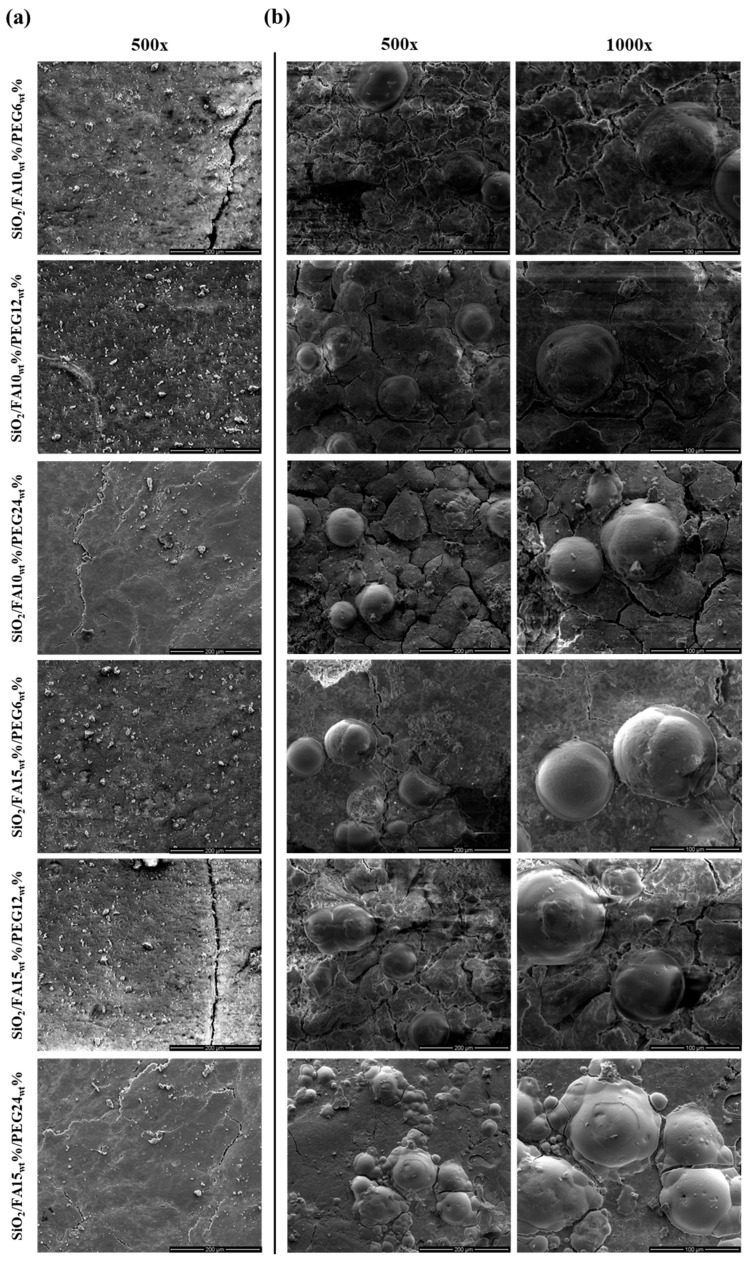
SEM images of SiO_2_/FA/PEG material (**a**) before and (**b**) after immersion in SBF for 21 days. Panels correspond to two different FA loadings (10_wt_% and 15_wt_%) and are presented at two magnifications (500× and 1000×).

Further investigation of HA formation was made with the SEM micrographs shown in Figure 5. In particular, images of the surface of all materials before immersion in SBF (Figure 5a) and after 21 days (Figure 5b) are reported. The micrographs clearly show the presence of globular aggregates distributed on the surface of the materials, likely due to HA nucleation. These spherical aggregates were observed in both formulations containing 10% and 15% by weight of natural drug, confirming that the incorporation of FA does not hinder apatite nucleation. At higher magnifications, the aggregates appear as compact globular structures, characteristic of the early stages of apatite crystallization on bioactive substrates. It was observed that the density of globular structure deposits was higher in the sample containing higher percentages of PEG by weight.

**Figure 6:**
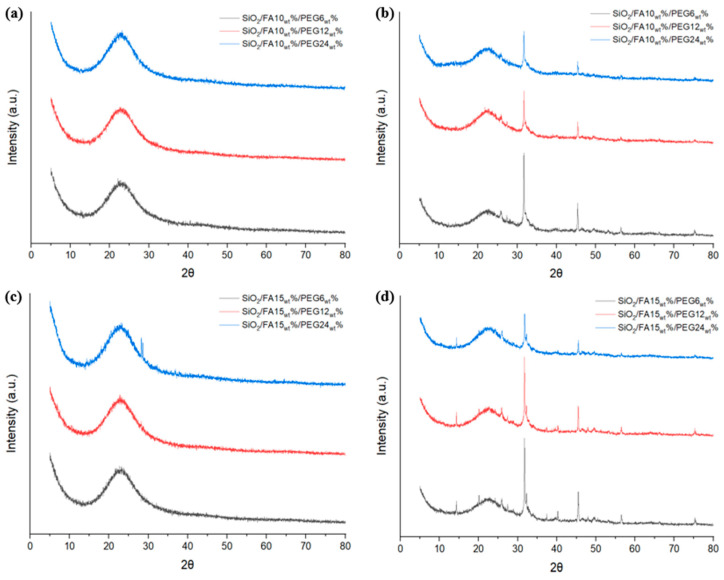
XRD graphs of SiO_2_/FA/PEG material (**a**,**c**) before and (**b**,**d**) after immersion in SBF for 21 days.

To confirm the presence of hydroxyapatite, XRD analysis was performed. Before immersion in SBF (Figure 6a,c), all samples exhibit a broad diffraction halo centered at approximately 20–25° (2θ), which is characteristic of an amorphous silica network. No sharp reflections attributable to crystalline FA or to any separate crystalline organic phase were observed, indicating that FA and PEG are molecularly dispersed within the hybrid matrix. Increasing PEG content (6–24_wt_%) produces only a slight increase in halo intensity and minor structuring in the same angular region, suggesting limited short-range ordering without the development of distinct crystalline domains.

After 21 days of immersion in SBF (Figure 6b,d), new sharp diffraction peaks appear superimposed on the amorphous background, mainly in the 26–32° (2θ) range. These peaks are consistent with the formation of crystalline calcium phosphate phases, typically associated with apatite deposition. Their presence confirms the bioactive behavior of the hybrid materials and the ability of the surface to induce nucleation and growth of a mineralized layer under physiological-like conditions. The intensity and sharpness of these peaks increase with PEG content, indicating that higher PEG loading promotes mineral nucleation and growth. Moreover, the 15_wt_% FA systems exhibit more pronounced apatite-related reflections compared to the 10_wt_% FA samples, suggesting enhanced mineralization at higher FA content. Importantly, in all immersed samples, the broad amorphous halo remains visible, indicating that the silica-based hybrid matrix retains its predominantly amorphous structure and that crystallization is mainly associated with the surface-deposited calcium phosphate layer rather than with structural rearrangements of the matrix itself.

## 3. Discussion

The obtained results indicate that the sol-gel process is effective for tailoring the properties of SiO_2_/PEG/FA hybrids through compositional control. In particular, PEG appears to act not only as an organic modifier but also as a component capable of influencing matrix flexibility and the kinetics of network formation, as suggested by the reduced rigidity of the materials and the longer gelation times at higher polymer contents. FA, beyond providing bioactive functionality, also contributes to the overall characteristics of the hybrid system. These observations support the view that both organic components actively participate in the formation of the final hybrid architecture, rather than being passively embedded within the silica matrix.

FTIR-ATR analysis was performed to assess the structural integration of FA and PEG into the sol-gel-derived silica matrix. The spectra of the starting materials (SiO_2_, PEG, FA) showed their characteristic functional groups: Si–O–Si stretching in SiO_2_ (~1080 cm^−1^), CH_2_ and C–O–C vibrations in PEG (~2900 cm^−1^ and ~1100 cm^−1^), and aromatic/C=O bands in FA (~1600–1450 cm^−1^ and ~1700 cm^−1^).

In the hybrid materials, new signals attributed to PEG and FA were clearly identifiable, confirming their successful incorporation into the silica network. The CH_2_ and C–O–C bands increased in intensity proportionally with PEG content, indicating good dispersion and interaction with the inorganic matrix. Meanwhile, minor bands associated with the aromatic ring of FA confirmed its presence, and the observed shift in the C=O band of FA confirmed a hydrogen-bonding interaction with silanol groups. No residual signals from unreacted precursors were detected, suggesting complete condensation and a homogeneous hybrid structure. The preservation of both organic and inorganic vibrational features supports the formation of an integrated, chemically compatible network. This confirms the effectiveness of the sol-gel route in producing tunable hybrid systems, where PEG and FA contribute to the modulation of structural, chemical, and potentially bioactive properties [21,22,23].

Surface analyses provided clear evidence of the composites’ bioactive behavior, with the ability to nucleate and grow apatite structure after immersion in SBF medium for 21 days. Indeed, sol-gel materials exhibit a surface rich in -OH groups, which are reactive with SBF salts. SEM micrographs revealed the presence of globular structures formed on the surfaces of the hybrid materials, particularly those with the highest weight percentage of PEG, attributable to the HA structure [24]. This finding indicates that, in addition to its activity as an inorganic compound, PEG also exhibits bioactive properties, thereby promoting the formation of apatite structure as its weight within the material increases. Moreover, the validity of these qualitative observations was substantiated by FTIR-ATR spectra, which demonstrated the progressive appearance and intensification of the phosphate bands characteristic of apatite with increasing PEG content [25]. The parallel increase in mineralization observed in both techniques indicates that PEG promotes not only the plasticizing effect of the material but also the direct formation of nucleation sites for apatite crystallization. The presence of 15_wt_% FA further amplified this effect, suggesting a synergistic role of FA and PEG in driving surface reactivity and bioactivity [26,27].

This increased bioactivity is reflected in the results of biological tests. Indeed, the tested formulations exhibited no indications of toxicity and were all able to maintain the viability of human dermal fibroblasts (HDF). At a concentration of 0.5 mg/mL, several composites exhibited a significant increase in cell viability in comparison with the control group. The most pronounced effects were observed in 15_wt_% FA systems containing 6–12_wt_% PEG, compared with systems containing a lower percentage of FA. This finding suggests that increasing the amount of natural drugs may improve the biological response of the hybrids under the tested conditions.

Indeed, FA is recognized for its antioxidant, anti-inflammatory, and protective properties against cellular oxidative stress [28,29,30].

Of particular pertinence is the analysis of gene expression, which revealed a marked upregulation of extracellular matrix-related genes (COL I and ELS), particularly in PEG-rich composites. Indeed, at elevated concentrations, PEG has been demonstrated to induce substantial alterations in the material’s hydration and composition, resulting in surfaces that exhibit reduced rigidity, reminiscent of the natural extracellular environment [31].

Several studies have indicated that a less rigid matrix of materials promotes a pro-synthetic phenotype in fibroblasts. This, in turn, has been shown to induce an increase in the synthesis and secretion of extracellular matrix components, including collagen genesis and elastin production [32,33].

The results obtained demonstrate a close correlation between the extent of globular structure formation and cellular response: the composites that showed greater mineralization potential also stimulated the strongest increase in HDF and matrix-related gene expression. The findings suggest that the optimal formulation for future biomedical applications is a combination of 12_wt_% PEG and 15_wt_% FA, achieving a balance between bioactivity and biocompatibility.

## 4. Materials and Methods

### 4.1. Sol-Gel Technique

**Figure 7:**
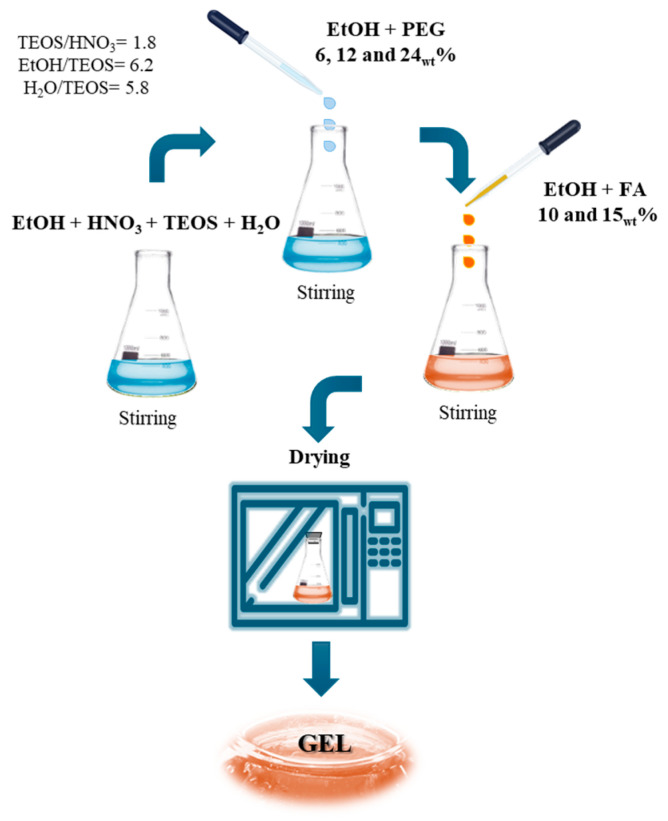
Flowchart of synthesis to obtain biomaterials.

SiO_2_-based hybrid materials containing varying percentages of PEG and FA were obtained through a sol-gel process at room temperature, starting from inorganic and organic precursors in solution. The synthesis flowchart is shown in Figure 7.

TEOS (Sigma-Aldrich, St. Louis, MO, USA) was used as the precursor for the inorganic phase, chosen for its high reactivity to hydrolysis under aqueous conditions. TEOS was dissolved in a mixture of EtOH (99.8%, Sigma-Aldrich) and distilled H_2_O. The solution was acidified with HNO_3_ (≥65%, Sigma-Aldrich) to catalyze the hydrolysis reaction. The molar ratios used were: TEOS/HNO_3_ = 1.8, EtOH/TEOS = 6.2, and H_2_O/TEOS = 5.8.

PEG (polyethylene glycol 400, Sigma-Aldrich), previously solubilized in ethanol, was added to this solution to improve dispersion, and finally, FA (≥99%, Sigma-Aldrich) was also dissolved in a small volume of ethanol.

Six different formulations were prepared, combining two weight percentages of FA (10 and 15_wt_%) with three increasing concentrations of PEG (6, 12, and 24_wt_%). After approximately 24–72 h of aging at 35 °C, depending on the composition, the formation of a transparent and homogeneous gel was observed. The gel was then left to dry at room temperature for 3, 5, and 7 days, respectively, for materials containing 6, 12, and 24_wt_% of PEG, until stable and glassy xerogels were obtained. After drying, the xerogels were ground into fine powders.

### 4.2. Attenuated Total Reflectance Spectroscopy

FTIR-ATR spectra were collected using a Perkin-Elmer FT-IR/NIR Spectrum 400 spectrophotometer equipped with an AIM-8800 infrared microscope (Shimadzu, Tokyo, Japan). The instrument was fitted with a built-in 3 mm-diameter germanium (Ge) FTIR-ATR semicircular prism, ensuring surface-sensitive analysis without additional sample preparation. For each material, pods of 100 mg were directly placed on the FTIR-ATR crystal for measurement. Spectra were recorded in the range of 500–4000 cm^−1^ with a spectral resolution of 4 cm^−1^.

### 4.3. Cytotoxicity Assay

Cytoxicity of tested materials was evaluated by MTT (3-(4,5-dimethyl-2-thiazolyl)-2,5-diphenyl-2H-tetrazolium bromide) assay on human immortalized fibroblasts (HDF) in vitro culture. The cells were grown in Dulbecco’s Modified Eagle Medium supplemented with 10% fetal bovine serum, 50.0 U/mL penicillin, and 100.0 μg/mL streptomycin, at 37 °C in a humidified atmosphere with 5% CO_2_. All the samples were tested at two concentrations: 0.5 and 1 mg/mL (*w*/*v*). In this context, 3 × 10^5^ cells/well were seeded in a standard 24-well plate, and eventual cytotoxicity was assessed after 48 h of incubation. After that, 200 μL of MTT solution (0.5 mg/mL) was added, and 3 h later, DMSO was employed to solubilize formazan salts. Relative optical densities were measured at 570 nm by a microplate reader (Tecan, Männedorf, Switzerland). Cellular viability was obtained by comparing the absorbance of each sample with that of untreated cells (CTR), shown as a percentage.

### 4.4. Real-Time Quantitative-PCR

All powder materials were dissolved in culture medium and assessed at a final 0.5 mg/mL concentration. 6 × 10^5^ cells/well were seeded in a standard 12-well plate and in vitro cultivated on biomaterials for 48 h. To evaluate the gene expression of type I collagen (COL I) and Elastin (ELS), real-time PCR analyses were performed. Total RNA was isolated by Trizol (Invitrogen, Thermo Fisher Scientific, Carlsbad, CA, USA) following the manufacturer’s instructions, and 1000 ng of each sample was reverse-transcribed (cDNA Synthesis Kit; Promega, Madison, WI, USA). Quantitative PCR analyses were carried out by using SYBR Green Master Mix (Roche, Basel, Switzerland) and LightCycler 480 instrument (Roche), and relative results were analyzed through LightCycler 480 software version 1.5.0.

### 4.5. Kokubo Test

Simulated body fluid (SBF) was used after previous preparation according to the method proposed by Kokubo and Takadama [21], which reproduces the ionic composition of human plasma. The following substances were dissolved in ultrapure water: 8.035 g/L of sodium chloride (NaCl), 0.355 g/L of sodium bicarbonate (NaHCO_3_), 0.225 g/L of potassium chloride (KCl), 0.231 g/L of potassium dihydrogen phosphate (K_2_HPO_4_·3H_2_O), 0.311 g/L of magnesium chloride (MgCl_2_·6H_2_O), 0.292 g/L of calcium chloride (CaCl_2_), 0.072 g/L of sodium sulfate (Na_2_SO_4_), and 6.118 g/L of 4-(2-hydroxyethyl)-1-piperazine methanesulfonic acid (HEPES). Dissolution was obtained at room temperature under constant stirring. 100 mg pods of each material were immersed in 25 mL of SBF at 37 °C for 21 days. The SBF solution was changed every two days following the protocol of Kokubo et al. The tablets were then placed in a desiccator for 24 h and analyzed by FTIR-ATR and SEM to evaluate the formation of HA on the surface.

### 4.6. Scanning Electron Microscopy (SEM)

The scanning electron microscopy (SEM) analysis was conducted using a FEI Quanta 200 FEG scanning electron microscope model (FEI Company, Eindhoven, Netherlands). The samples were prepared by mounting pods of the material onto aluminum stubs using carbon tape. The samples were gold-sputtered for 180 s and imaged under high vacuum. Images were captured for the purpose of assessing surface morphology at magnifications of 500× and 1000×. The data were acquired and processed using the software provided by the manufacturer.

### 4.7. X-Ray Diffraction (XRD)

X-ray diffraction (XRD) analysis was performed using a Bruker D8 Advance diffractometer (Bruker AXS, Karlsruhe, Germany) operating in θ–2θ configuration at the Instituto Universitario de Investigación de Tecnología de Materiales (IUTM), Universitat Politècnica de València, Alcoy, Spain. Data were collected in the 2θ range of 5–80° with a step size of 0.02° and a counting time of 1 s per step. Powder samples were analyzed at room temperature.

## 5. Conclusions

Silica-based sol-gel hybrid materials containing different percentages of PEG and FA have shown promise for biomedical applications, thanks to a favorable combination of chemical, physical, biological, and bioactive properties.

FTIR-ATR analysis confirmed the successful incorporation of organic components within the inorganic SiO_2_ matrix. In particular, the increase in intensity of the PEG ether bands (C–O–C) with PEG loading reflected the increasing polymer content, while the slight shift of the FA carbonyl band suggested hydrogen-bonding interactions with silanol groups.

Biocompatibility testing using the MTT assay demonstrated good tolerability of the materials, with cell viability levels above the cytotoxicity threshold. The addition of FA did not compromise cell viability; in fact, in samples with 15_wt_% FA, a slight stimulation of metabolic activity was observed, likely attributable to the known antioxidant properties of FA. Gene expression analysis showed that all samples increased gene expression of both COL I and ELS compared to CTR, particularly the materials with 15_wt_% FA.

Regarding bioactivity, the formation of calcium phosphates on the surfaces of samples immersed in simulated physiological fluid (SBF) was confirmed by FTIR-ATR, with the appearance of peaks typical of the phosphate group (approximately 560 and 600 cm^−1^) and SEM myographs. The formation of a globular structure was observed more frequently in materials with high percentages of PEG by weight (24_wt_%).

Finally, the results obtained indicate that the combination of 12_wt_% PEG and 15_wt_% FA represents an optimal formulation in terms of the balance between bioactivity and biocompatibility for future biomedical applications.

## Figures and Tables

**Table 1:** Products obtained from sol–gel synthesis.

Label	System Composition		
	Inorganic Matrix SiO_2_ (_wt_%)	Organic Matrix FA (_wt_%)	Polymeric Matrix PEG (_wt_%)
SiO_2_	100	---	---
SiO_2_/FA10_wt_%/PEG6_wt_%	84	10	6
SiO_2_/FA10_wt_%/PEG12_wt_%	78	10	12
SiO_2_/FA10_wt_%/PEG24_wt_%	66	10	24
SiO_2_/FA15_wt_%/PEG6_wt_%	79	15	6
SiO_2_/FA15_wt_%/PEG12_wt_%	73	15	12
SiO_2_/FA15_wt_%/PEG24_wt_%	61	15	24

## Data Availability

The original contributions presented in this study are included in the article. Further inquiries can be directed to the corresponding author.

## Abbreviations

The following abbreviations are used in this manuscript:

FA	Ferulic acid
TEOS	Tetraethyl orthosilicate
PEG	Polyethylene glycol
FTIR	Fourier-transform infrared
ATR	Attenuated total reflectance
SBF	Simulated body fluid
MTT	(3-(4,5-dimethyl-2-thiazolyl)-2,5-diphenyl-2H-tetrazolium bromide)
ELS	Elastin
COL I	Type I collagen
HA	Hydroxyapatite
